# Bibliometric Analysis of Poor Oral Health as a Risk Factor for Oral Cancer

**DOI:** 10.7759/cureus.36015

**Published:** 2023-03-11

**Authors:** Amit V Mahuli, Vidya Sagar, Vedha VPK, Simpy A Mahuli, Anit Kujur

**Affiliations:** 1 Public Health Dentistry and Preventive Dentistry, Dental College, Rajendra Institute of Medical Sciences, Ranchi, IND; 2 Preventive and Social Medicine, Rajendra Institute of Medical Sciences, Ranchi, IND; 3 Public Health Dentistry, Rajendra Institute of Medical Sciences, Ranchi, IND

**Keywords:** citation index, risk factors, poor oral health, oral cancer, bibliometrics

## Abstract

Poor oral health is a risk factor for oral cancer, and bibliometrics can tell us important things about publication trends and research. Oral cancer risk factors include smoking, betel nut chewing, alcohol consumption, trauma from sharp teeth, chronic infections, and other factors related to oral health. There is a need to understand the role of poor oral health as a risk factor. Thus, this study aimed to conduct a bibliometric analysis of the literature on poor oral health as a risk factor for oral cancer. A bibliometric analysis was conducted for poor oral health as a risk factor for oral cancer using RStudio 2021.09.0+351 “Ghost Orchid” Release (2021-09-20) for Windows, package “bibliometrix.” The literary data for this study were derived from Elsevier's Scopus database, and the data were exported in BibTex format. The results considered the time frame of 1983 to 2022, with journals, books, newspaper articles, and others as sources, accounting for a total of 543 documents. The search yielded a total of 2,882 authors, with a total of 3,306 appearances. The results show that the research on poor oral health and oral cancer is mainly led by the United States (106), India (49), and China (46). The top author is Warnakulasuriya S, followed by Worthington HV. The research shows the countries that are currently working on the topics and helps set up future collaborations to improve the evidence produced and help the scientific community by finding research gaps and experts in this area of research.

## Introduction and background

The International Classification of Diseases Tenth Edition followed by the World Health Organization has given lip and mouth cancer the code C00-06. The estimated number of cases coded C00-06 as per Globocan 2020 in both genders is 377,713, with a mortality of 177,757, with males reporting twice the number of cases and mortality in comparison to females. South-central Asia alone contributed to 174,448 cases and 98,015 mortalities [1]. In the Indian subcontinent, oral cancer and potentially malignant diseases are major public health concerns. Oral cancer is associated with tobacco use as a significant risk factor. Multiple factors have shown an association with oral cancer. Many studies have reported the role of oral hygiene (tooth brushing), gum disease, the number of missing teeth, the use of dentures, and even regular visits to the dental clinic as related factors in head and neck cancers (lip, oral cavity, and pharynx C00-C14), including cancer of the lips and oral cavity [2-5].

The amount of research done on this topic and the number of studies that investigate the role of oral health-related risk factors in oral cancer need to be investigated. Bibliometric analysis is used for various purposes, including identifying new patterns in articles and journals, ways of collaboration, and research elements, as well as investigating the intellectual framework of a specific area in the existing literature [6]. Bibliometrics of poor oral health as a risk factor for oral cancer can provide vital information on publication trends. Other oral cancer risk factors are well documented and appear as major risk factors contributing to the disease. There is a need to comprehend the role of oral health-related risk factors in oral cancer and understand the research areas, centers, and authors contributing to the research on the topic [7]. Thus, this study aimed to conduct a bibliometric analysis of the literature on poor oral health as a risk factor for oral cancer.

## Review

Methodology

Bibliometrics is the primary tool of science used for quantitative analysis of publications, statistics for journal articles, and citation counts. Today, quantitative analysis of publication and citation data is used in almost all scientific fields to measure a community’s growth, maturity, top authors, conceptual and intellectual maps, and trends [6]. This study used performance analysis and science mapping, including publication-related metrics, citation-related metrics, citation analysis, co-citation, co-wording, and co-authorship analysis [8].

Data Extraction

The data were extracted from Elsevier’s Scopus database. The data were searched using the keywords “poor oral health and oral cancer.” The search was done under documents, all necessary fields were selected, and data were exported in BibTex format.

Data Analysis

The data were analyzed using RStudio 2021.09.0+351 “Ghost Orchid” Release (2021-09-20) for Windows, package “bibliometrix” [8]. The data extracted were run through the R program using the commands in the bibliometrix package installed in R [9]. The document type, keywords and author keywords, author collaboration, annual scientific production on the topic, most productive authors, citations, author country, published sources, author dominance factor, h-index, g-index, m-index, and other essential variables were examined.

Results

The results considered the time frame 1983-2022 with sources (e.g., journals, books, etc.) are listed in Table 1.

**Table 1:** Various document types in the analyzed data.

Document types	
Article	412
Article in press	1
Book chapter	2
Conference paper	8
Letter	3
Note	2
Review	113
Short survey	2

**Figure 1:**
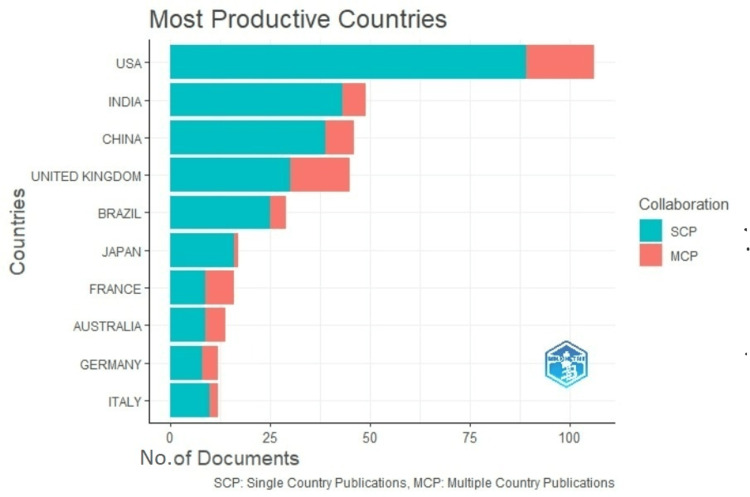
Most productive countries that published on poor oral health and oral cancer.

The topic showed an annual growth rate of 9.81% and 33.72% average citations per document. A total of 2,882 different authors appeared in the search and their total appearances were 3,306. Single-authored documents were 38, co-authored documents were 6.09, and documents with international co-authors were 20.99%. The results clearly show that the research on poor oral health and oral cancer is mainly led by the United States (106), India (49), and China (46) (Figure 1).

**Figure 2:**
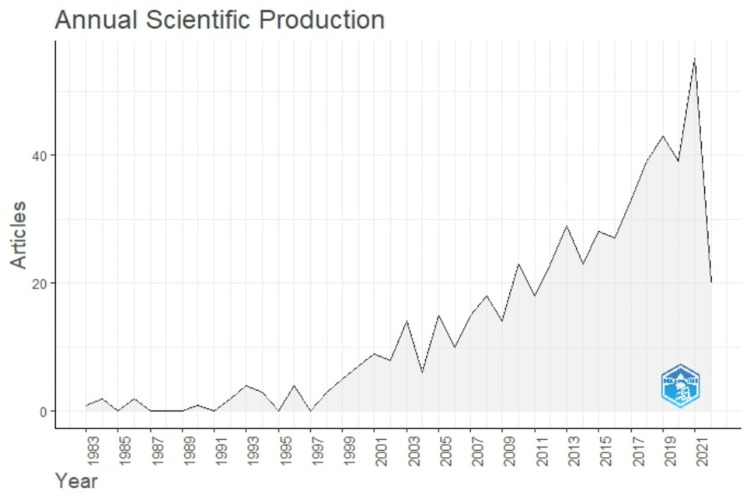
Annual scientific production of the literature on the topic.

Annual scientific production of the literature showed a peak in 2021 with 55 documents (Figure 2).

**Figure 3:**
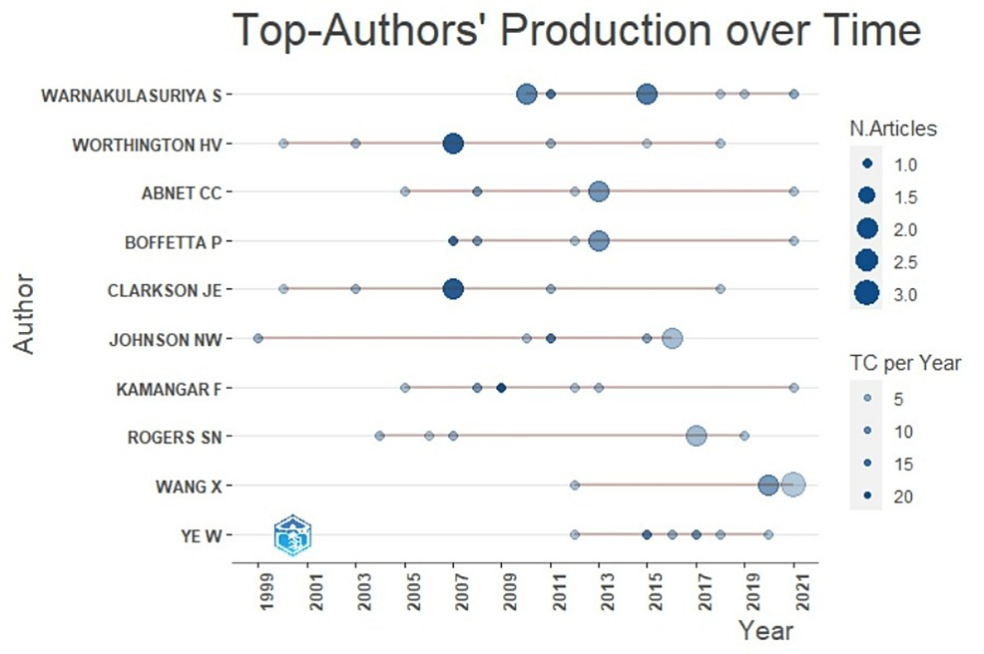
Top authors’ production of the literature on the topic over time.

Warnakulasuriya S, Worthington HV, and Abnet CC were the top authors of the published literature (Figure 3).

**Figure 4:**
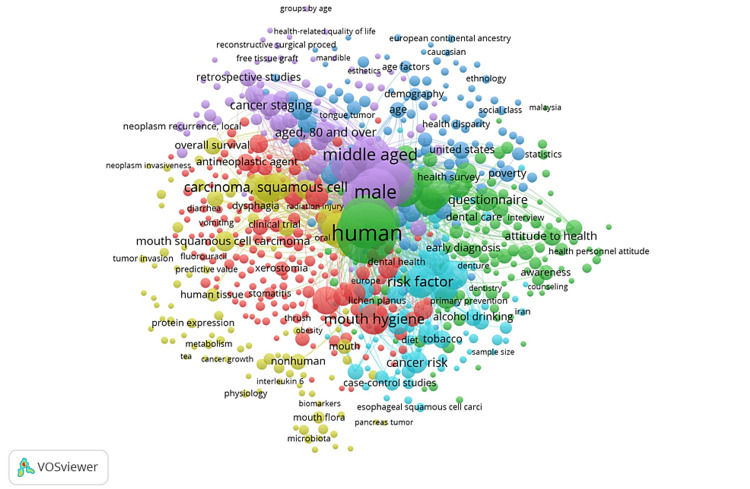
Keyword co-occurrences in the literature analyzed.

The country collaboration in the publication showed the United States, India, China, and the United Kingdom as the major collaborators. The keywords co-occurrences in the literature show squamous cell carcinoma, mouth, human, risk factor, mouth disease, gender, age, and other keywords co-occurrences (Figure 4). The co-occurrences were analyzed using VOSviewer 1.6.18.

The United States earned the maximum number of citations (4,049), followed by the United Kingdom (2,602), whereas India was in the ninth position with 433 (Table 2).

**Table 2:** Total citations per country.

Country	Total citations	Average article citations
United States	4,049	38.20
United Kingdom	2,602	57.82
Switzerland	2,282	456.40
Brazil	1,041	35.90
China	912	19.83
France	692	43.25
Canada	535	53.50
Finland	447	89.40
India	433	8.84
Malaysia	356	44.50

The Oral Oncology journal (36), followed by Supportive Care In Cancer (14), and the Asian Pacific Journal of Cancer Prevention (13) were the three top journals that published the literature on this topic (Table 3).

**Table 3:** Most relevant sources (journal) publishing on the topic.

Sources	Articles
Oral Oncology	36
Supportive care in Cancer	14
Asian Pacific Journal Ccancer Prevantion	13
PLoS One	12
Community Dentistry and Oral Epidemiology	8
British Dental Journal	7
Cancer	7
Cancers	7
Head and Neck	7
Oral Diseases	7

The author dominance factor indicated that most single-authored publications were of Chen X (0.4) (Table 4).

**Table 4:** Author dominance factor (DF).

Author	Dominance factor	Total articles	Single-authored	Multi-authored	First-authored	Rank by article	Rank by DF
Chen X	0.4	5	0	5	2	9	1
Clarkson JE	0.33333	6	0	6	2	3	2
Johnson NW	0.33333	6	0	6	2	3	2
Rogers SN	0.33333	6	0	6	2	3	2
Worthington HV	0.28571	7	0	7	2	2	5
Chand JS	0.25	4	0	4	1	10	6
Abnet CC	0.16666	6	0	6	1	3	7
Kamangar F	0.16666	6	0	6	1	3	7
Wang X	0.16666	6	0	6	1	3	7
Warnakulasuriya S	0.125	8	0	8	1	1	10

The h-index, g-index, m-index, total citation (TC), net production (NP), and starting year (PY_start) are shown in Table 5.

**Table 5:** h-index, g-index, m-index, total citation (TC), net production (NP), starting year (PY_start).

Element	h-index	g-index	m-index	TC	NP	PY_start
Abnet CC	5	6	0.277778	290	6	2005
Boffetta P	5	6	0.3125	494	6	2007
Clarkson JE	6	6	0.26087	455	6	2000
Johnson NW	6	6	0.25	383	6	1999
Kamangar F	5	6	0.277778	515	6	2005
Rogers SN	6	6	0.31579	193	6	2004
Wang X	2	6	0.181818	54	6	2012
Warnakulasuriya S	7	8	0.538462	448	8	2010
Worthington HV	7	7	0.304348	465	7	2000
Ye W	6	6	0.545455	244	6	2012

Discussion

This study examined how much research has been done on oral cancer and poor oral health worldwide using a keyword search on the Scopus database. Scopus is the largest database of peer-reviewed literature, including books, journals, and conference proceedings. Every year, the journals in the Scopus database are examined to ensure that they meet high standards. Scopus also provides information about authors, such as their affiliations, a list of their publications with bibliographic information, references, and the number of times each published work has been cited. As a result, the Scopus database was employed in the current investigation to retrieve information [10,11].

The maximum number of research publications on oral cancer and poor oral health was published in 2021. The United States, India, and China are the leaders in publications on this topic. The United States is the most developed center for cancer registries and early cancer detection of oral cancer, which impacts the survival rate of the patients, thus aiding in the maximum research output in publications [12]. India has the highest oral cancer prevalence, causing many centers of prominence to conduct research on this topic [13]. National Cancer Registry Programme in India is run by the Indian Council of Medical Research in leading sites such as Bangalore, Delhi, Mumbai, Chennai, Bhopal, Ahmedabad, Barshi, and others [14]. Increased oral cancer prevalence has led to increased research and reporting from China [15].

The countries showing the highest citations earned for publications are the United States, United Kingdom, and Switzerland, followed by other nations, with India in the ninth place. Although India is the second country with the most research papers published, it has the fewest citations. This is most likely because articles were published in low-impact journals because of a lack of funding [16]. The many organizations that can fund oral cancer research should be connected to the centers and researchers of eminence in the field [16,17]. The author dominance factor indicates the first authored publications of the top 10 authors to their total publications [8,9].

Warnakulasuriya S was the most cited and top author with publications, and Chen X led the author dominance index with the most publications as the first author. Researchers use citation tracking to find the most critical articles on a subject and how often their published papers are cited. Citations indicate the genuine acknowledgment of work and the short-term, observable impact on global research. The present study also shows the best citations earned based on country, author, and journal, enabling researchers to focus on global trends and critical research on poor oral health and oral cancer [16,17]. The h-index of the author highlights the number of papers with citations number higher or equal to h [18].

The h-index and the g-index are mainly influenced by the number of publications an author publishes. The g-index gives more weightage to highly cited articles in comparison to the h-index. The m-index is another type of h-index that highlights the h-index since the first publication [19]. The bibliometric searches include the related terms and might not produce specific results but show the overall progress, production, and trend on the topic of interest. The present bibliometric analysis has the limitation of considering one database for bibliometrics.

## Conclusions

The research shows the countries that are currently working on the topics and helps set up future partnerships to improve the evidence and help the scientific communities. Researchers can learn from authors who are most often cited and have published the most. The countries can review the literature produced and the citations generated over a period of time and plan to fill gaps in research funding, thus improving the quality of research. Poor oral health can be seen as one of the major risk factors for oral cancer, and bibliometric analysis can help fill these research gaps.
